# Multi-Component 3D Bioprinted Platform with Sacrificial Matrix and Collagen-Based Bioinks for Skeletal Muscle Tissue Engineering

**DOI:** 10.3390/polym18101223

**Published:** 2026-05-17

**Authors:** Carmen Mª. Granados-Carrera, Francisco José Calero Castro, Victor M. Perez-Puyana, Mercedes Jiménez-Rosado, Jaime Navarrete-Damián, Fernando de la Portilla de Juan, Alberto Romero

**Affiliations:** 1 Departamento de Ingeniería Química, Facultad de Química, Universidad de Sevilla, 41012 Sevilla, Spain; cargracar@alum.us.es (C.M.G.-C.); alromero@us.es (A.R.); 2 Hospital Universitario “Virgen del Rocío”, IBiS, CSIC/Universidad de Sevilla, 41013 Sevilla, Spain; fjcalerocastro@gmail.com (F.J.C.C.); fportilla@us.es (F.d.l.P.d.J.); 3 Departamento de Cirugía General y Digestiva, Hospital Universitario “Virgen del Rocío”, IBiS, CSIC/Universidad de Sevilla, 41013 Sevilla, Spain; 4 Departamento de Ingeniería y Ciencia de los Materiales y del Transporte, Universidad de Sevilla, 41092 Sevilla, Spain; 5 Grupo de Ingeniería Química, Ambiental y Bioprocesos, Instituto I4, Universidad de León, 24071 Leon, Spain; mjimr@unileon.es; 6 Departamento de Ingeniería Industrial, Instituto Tecnológico de San Juan del Río, Queretaro 76800, Mexico; jaime.nd@sjuanrio.tecnm.mx

**Keywords:** 3D bioprinting, skeletal muscle tissue engineering, rheology, collagen-based bioinks, extracellular matrix, scaffolds, biofabrication

## Abstract

The development of biomimetic and mechanically functional constructs remains a major challenge in skeletal muscle tissue engineering. In this study, we present a multi-component 3D bioprinted platform integrating a polycaprolactone (PCL) support for mechanical stimulation, a sacrificial gelatin (GE) matrix for controlled bioink deposition, and collagen-based bioinks laden with *Rattus norvegicus* L6 skeletal muscle cells. The influence of PCL architecture, GE concentration (0.75, 1.5 and 3 wt%), and bioink composition—collagen (C), collagen–Matrigel (CM), and extracellular matrix-based (ECM)—was systematically evaluated. Rheological characterization demonstrated that all bioinks exhibited shear-thinning behavior and suitable viscoelastic properties for extrusion-based bioprinting, with sufficient mechanical stability to withstand dynamic bioreactor conditions. Microstructural analysis revealed highly interconnected porous networks, particularly in ECM-based scaffolds. While no statistically significant differences were observed, the ECM-based bioinks showed the highest cell viability and improved structural organization. Overall, this work demonstrates a versatile bioprinting strategy that combines mechanical support and biomimetic environments, highlighting the potential of ECM-based bioinks for the fabrication of functional skeletal muscle constructs.

## 1. Introduction

Skeletal muscle (SM) tissue is one of the most abundant tissues in the complex human anatomy (approximately 45% of body mass), performing essential functions such as voluntary movement or energy metabolism. However, its regenerative capacity is limited following traumatic injuries, tumor resections or degenerative diseases, which can lead to chronic loss of muscle function at various levels [1,2]. Consequently, this situation generally results in significant muscle loss, which cannot be naturally recovered, highlighting the need for skeletal muscle regeneration. Among the alternatives that have been used are strategies such as surgery, medicine, physical therapy, and cell therapy [2,3]. However, in recent years, there has been particular interest in addressing this issue through tissue engineering (TE) [4,5,6].

Specifically, TE is an innovative interdisciplinary approach to developing biofunctional scaffolds [7,8] that provide three-dimensional support to promote cell growth by an appropriate microstructure and cellular signaling through proper cell–scaffold and cell–cell interactions [9,10]. In this context, the extracellular matrix (ECM) plays a fundamental role in the structural support and biochemistry of cellular processes (including adhesion, proliferation, migration, and differentiation) [11,12,13,14,15]. Hence, the scaffold’s main purpose is the obtention of a biomimetic structure that emulates a functional muscle graft for skeletal muscle tissue engineering (SMTE) [16]. Traditional biofabrication techniques, including lyophilization or electrospinning, among others, have been employed [16], although they are not able to reproduce SM tissue structure at the clinical scale, making this one of the primary challenges currently facing the field [17,18,19].

Consequently, more advanced techniques are needed to enable greater control over cell deposition processes and the development of biomaterials themselves. Among the most notable biofabrication techniques in recent decades is three-dimensional (3D) bioprinting, which is characterized by enabling: (i) the use of a variety of raw materials (synthetic and natural polymers); (ii) the combination with different additives such as biomolecules, drugs, and nanomaterials; (iii) ease of the manufacturing process; (iv) repeatability; (v) the ability to control the microenvironment; and (vi) cell affinity [20,21]. Specifically, 3D bioprinting has garnered attention due to the creation of soft, hydrated and customized structures with predefined pore volumes and anisotropic cellular organization, combining the ability to encapsulate cells and drugs with the achievement of a high degree of in vivo mimicry [22,23,24] with SM tissues, resulting from the creation of a highly interconnected and organized hierarchical fibrous architecture with sufficient vascularization and innervation [17,25,26]. 

Indeed, a large number of synthetic polymers have been widely used due to their favorable mechanical properties and printability, including examples such as polycaprolactone (PCL), polylactic acid (PLA), or polyglycolic acid (PGA) [27,28,29,30,31]. However, their low bioactivity makes it necessary to combine them with natural polymers (also known as biopolymers) such as collagen, gelatin, hyaluronic acid, or alginate, in order to improve cell adhesion and biological performance [32,33,34,35,36,37]. For instance, Navaei et al. (2021) [38] fabricated composite scaffolds through the combination of 3D printing and freeze-drying techniques, which combined mechanical strength, flexibility, biocompatibility, biodegradability and the possibility of monolayer cell adhesion. In the same way, Lee et al. (2024) [21] combined poly(ethylene glycol) and a copolymer of polycaprolactone for the 3D bioprinting of biodegradable hydrogel scaffolds.

In particular, in SMTE, collagen-based bioinks have been widely employed in biomedical applications due to their ability to adhere to cells, as well as their biocompatibility, biodegradability, and similarity to ECM components [26,39,40,41]. Thereby, their utilization possibilities include modulating the mechanical properties of the scaffolds and forming aligned muscle fibers, thereby contributing to tissue maturation [42,43]. Particularly, its concentration affects the porosity of the scaffold, which is directly linked to the required nutrient diffusion and affects the adequacy of the printing process [44]. Moreover, other collagen derivatives, such as gelatin, possess excellent biopolymeric properties and are adequate as sacrificial biomaterials because of their temperature-reversible properties, thereby enhancing the porosity of the constructs [42,43,45]. For instance, Gong et al. (2025) [46] employed collagen showing an adequate response (physiologically, bioactivity, and functionally), although it was necessary to incorporate other bioactive compounds.

Previous studies have demonstrated that composite hydrogels incorporating collagen with other biomolecules, such as fibrin, hyaluronic acid, or Matrigel, can support myoblast differentiation and the formation of aligned muscle fibers [39,40], thereby increasing the complexity of the scaffold and being more similar to the complex ECM. Specifically, depending on the concentration employed, Matrigel can serve as a biological supplement to the collagen, fomenting the formation of a fibrillar architecture with increased structural complexity and reduced porosity that favors cell proliferation [47,48] or, in low concentrations, as a growth factor [49]. Therefore, to mimic the complete physiology of living tissue, other compounds can be incorporated in the formulations, such as fibrinogen, which facilitates the crosslinking, improving the viscosity of the bioink and playing an essential role in tissue healing [26,50], or bovine serum albumin that provides a similar osmotic pressure environment to blood plasma, hence protecting the cells during the printing process, preventing fouling, and promoting ink flow [51]. Despite these advances and complex structures, current approaches still face significant limitations, including insufficient mechanical stability, poor control over bioink deposition, and limited integration between structural support and biological functionality [52,53,54,55].

In this regard, the integration of mechanically robust support with biomimetic bioinks and sacrificial matrices represents a promising yet underexplored strategy to improve scaffold performance under dynamic culture conditions. This aspect is particularly relevant in SMTE, where mechanical stimulation plays a crucial role in regulating cell alignment, differentiation, and maturation [56]. Therefore, scaffolds must not only provide a suitable biochemical environment but also effectively transmit mechanical cues to the embedded cells [57]. In this context, the use of sacrificial materials can facilitate precise bioink deposition while enabling subsequent structural rearrangement and cell growth. However, the combined effect of support architecture, sacrificial matrix properties, and bioink composition on the mechanical and biological performance of bioprinted constructs remains poorly understood, being the focus of outgoing research. 

Therefore, this work aims to develop and evaluate a multi-component 3D bioprinted platform for skeletal muscle tissue engineering. The proposed system integrates: (i) a PCL support to provide mechanical stability and enable load transmission, (ii) a gelatin-based sacrificial matrix to assist bioink deposition and (iii) collagen-based bioinks incorporating L6 skeletal muscle cells. The influence of support design, matrix composition, and bioink formulation on the rheological, microstructural, and biological properties of the constructs is systematically investigated. This study provides new insights into the design of mechanically functional and biologically active bioprinted systems for advanced tissue engineering applications.

## 2. Materials and Methods

### 2.1. Materials

Low-molecular-weight polycaprolactone (PCL, 50,000 g/mol; 3D4MAKERS, Haarlem, The Netherlands) was used to fabricate the supporting structures. Gelatin (type I, Bloom index 80–120, protein content > 95%; HENAN BOOM GELATIN CO LTD, Zhengzhou, China) was employed as the sacrificial matrix material. Gelatin solutions were prepared using 0.05 M acetic acid (Panreac Química SA, Barcelona, Spain; pH 3.2) as the solvent.

Bioinks were formulated using type I collagen (pH 2–3, purity > 99.9%; Cell Guidance Systems, Cambridge, UK). Additional components included Matrigel (Corning, Corning, NY, USA), fibrinogen (MP Biomedicals, Shanghai, China), bovine serum albumin (BSA), and thrombin (Sigma Aldrich, Darmstadt, Germany).

### 2.2. Processing of the Matrix

The sacrificial matrix serves as a platform for the subsequent deposition of bioinks. Gelatin was used as the sacrificial matrix, where the bioinks maintain the shape until crosslinking occurs, as described in previous studies [58]. Different matrices were prepared at 0.75, 1.5 and 3.0 wt% using acetic acid (0.05 M) as the solvent in a stirred vessel and gelled for 24 h at 4 °C.

### 2.3. Processing of the Bioinks

Three collagen-based bioinks (scaffolds) with different compositions were prepared. The first (labeled C) was made with type I collagen alone at a concentration of 4 mg/mL. The second (labeled CM) was a mixture of type I collagen and Matrigel at concentrations of 3 mg/mL and 2.5 mg/mL, respectively. Finally, another bioink was prepared with ECM components consisting of type I collagen at 3 mg/mL, Matrigel at 0.3125 mg/mL, fibrinogen at 12.5 mg/mL, and bovine serum albumin at 12.5 mg/mL (referred to as ECM).

Scaffolds were cultured using a Rattus norvegicus L6 skeletal muscle cell line (ATCC^®^ CRL-1458) in a growth medium plate containing high-glucose Dulbecco’s modified Eagle’s medium (SH30285.01, HyCloneTM, Fisher Scientific, Pittsburgh, PA, USA) supplemented with 10% fetal bovine serum (F7524, Sigma Aldrich, Darmstadt, Germany) and 1% P/S (15140-122, Gibco, New York, NY, USA) at 37 °C in a 5% CO_2_ atmosphere. After 1 week, MG was replaced with differentiation medium containing Dulbecco’s modified Eagle’s medium high glucose (SH30285.01, HyCloneTM, Fisher Scientific, Pittsburgh, PA, USA) supplemented with 2% fetal bovine serum (F7524, Sigma Aldrich, Darmstadt, Germany) and 1% P/S (15140-122, Gibco) until 4 weeks after bioprinting. The medium was changed regularly every 48-72 h. Finally, myoblasts were encapsulated in the bioplates at a concentration of 20 × 10^6^ cells/mL, and the encapsulated bioplates were transferred to an imprinting syringe.

### 2.4. Multidevice 3D Design and Printing

The device consists of a collagen-based scaffold placed on a gelatin matrix, which in turn is deposited onto PCL support. The function of the support is to serve as an anchoring platform for the assembly formed with the bioinks and to help the scaffold maintain its structural integrity when the cells are subjected to periodic mechanical stresses in the bioreactor. Two 3D support designs were created using FreeCAD 0.17 software [42]. The first support (Figure S1a) consisted of a 20 × 10 mm structure with clamping jaws (3 × 10 mm) attached to the end of the support by a rhombus (named as configuration A). The second support (Figure S1b) was 20 × 5 mm with 3 × 5 mm jaws (named as configuration B). Both supports were 0.8 mm thick. The innovative design of the scaffold and support has been carried out considering a geometry that enables stable deposition of the gelatin matrix and bioink printing. Furthermore, the design of the clapping jaws was carried out to improve the subsequent mechanical stimulation that the system would withstand in the bioreactor. Hence, a dimension of 20 mm was chosen to match the characteristic size of the muscle tissue construct as reported in the literature [20,59]. Moreover, a wider configuration of 10 mm was employed to give a sufficient area of contact while reducing stress concentration and minimizing slippage [60]. However, the reduced width (5 mm) was used to compare with the other configuration to evaluate the effect on tension distribution and tissue failure [61].

Figure S2 shows a schematic diagram of the fabrication steps of the 3D bioprinted multidevice (support, matrix and scaffold). Prior to 3D printing, the laminar flow cabinet (N-Biotek Bio-workstation Beauty Cell NB-803 MSF, AEMA^®^, Bucheon-si, Gyeonggi-do, Korea) was sterilized for 40 min together with the 3D printer by UV irradiation. The PCL-based supports were then printed (Figure S2a). After PCL printing, 500 µL of gelatin was deposited and served as a bath for crosslinking the bioinks (Figure S2b). The nozzle of this syringe was 0.4 mm. Subsequently, the pH was modified with 100 µL of neutralizing solution that was deposited over the gelatin with the second syringe (Figure S2c). For crosslinking of the ECM bioink, neutralizing solution in combination with thrombin at 0.01 U/mL (T7326, Sigma Aldrich, Darmstadt, Germany) was used for fibrinogen coagulation that was mixed with neutralizing solution and was deposited with the second syringe. The nozzle of this syringe was 0.2 mm. Finally, 500 µL of the bioink was deposited (Figure S2d) and heated to 37 °C to promote crosslinking of the bioink and liquefaction of the gelatin matrix (Figure S2e).

### 2.5. Characterization

The supports and scaffolds were rheologically characterized using a Dynamic Mechanical Analyzer (DMA), model RSA3 rheometer (TA Instruments, New Castle, DE, USA). Dynamic compression tests were performed on the scaffolds with a plate–plate geometry, and a three-point flexure test was conducted on the support. Two different dynamic tests were performed on both specimen types (oscillatory tests), strain sweep and frequency sweep tests, in which the elastic and viscous moduli were denoted as E′ and E″, respectively. First, a strain sweep test was carried out from 2–10^−4^ to 2% at a constant frequency of 1 Hz. Its purpose was to identify the linear viscoelastic region and determine the critical strain (γ_c_). In addition, frequency sweep tests were performed from 0.2 to 20 Hz at constant strain within the linear viscoelastic range. Furthermore, in the case of scaffolds, these tests were initially performed: (i) at 22°C, which was the selected bioprinting temperature, i.e., an ambient temperature, to maintain suitable cell availability and functionality during the printing process, as well as avoid a rapid gelation [62]; and (ii) at 37°C, which is the application temperature, simulating the standard temperature of human body which is the desired environmental for SMTE applications [63].

The elastic modulus (E′) and complex viscosity (η*) (Equation (1)) of the systems were tabulated at 5 Hz (named as E′_5_ and η*_5_, respectively) to better compare and understand the behavior of the scaffolds in the bioreactor since it operates at a similar frequency (around 4.7 Hz cycles) [64].

(1) $$\eta^{*}={\left[{\left(\frac{E^{\prime }}{w}\right)}^{2}+{\left(\frac{E^{\prime \prime }}{w}\right)}^{2}\right]}^{1/2}$$

The rheological properties of the different matrices were determined by dynamic shear tests using an AR 2000 rheometer (TA Instrument, USA). Rough aluminum plates (diameter: 60 mm) were used to minimize potential inertia and prevent slippage. In addition, temperature control was maintained by a Peltier device connected to a thermostatic bath. Specifically, strain sweep tests were performed to achieve a strain value within the linear viscoelastic range (LVR), followed by frequency sweep tests at a constant strain within this range. The shear elastic modulus (G′) and shear viscous modulus (G″) values were derived from these measurements. Flow curves ranging from 1 to 500 Pa were then generated to evaluate the flow characteristics of the matrices through the syringe of the 3D bioprinter. The experiments were performed at the bioprinting temperature (22 °C). Finally, critical temperature ramps were performed to determine the temperature range at which the matrix lost its structural integrity. These evaluations were performed at a constant strain of 2% and 1 Hz.

The morphological characteristics of the components of the multidevice were evaluated using cryo-scanning electron microscopy (cryo-SEM) with a Zeiss EVO model (Zeiss, Oberkochen, Germany) equipped with a secondary electron detector operating at an accelerating voltage of 10 kV. This technique was used because the hydrogel matrix and bioinks have a moisture content exceeding 90%, rendering conventional scanning electron microscopy inadequate for preserving their structure, necessitating a cryogenic fixation method [65]. To achieve this, the hydrogels were immersed in a liquid nitrogen bath, followed by sputtering with gold and platinum [66].

For cell viability assays, scaffolds were fixed after 4 weeks of culture in 4% PFA (paraformaldehyde) for immediate embedding in paraffin blocks. Sections approximately 4 µm thick were obtained using a microtome. Staining was performed with hematoxylin (GHS316 500ML: Hematoxylin solution, Gill no. 3) and eosin (HT110116 500ML: Eosin Y solution). Five images of each sample were taken with the Olympus BX-61 optical microscope at “10×” magnification to obtain a representative image of the scaffold showing biofouling, nuclei and cell cytoplasm. The FIJI version of ImageJ software (version 1.54g) was used to analyze the microphotographs and to measure cell population and cell viability [67]. Cell population was defined as the number of live cells detected. Equation (2) was used to measure cell viability:

(2) $$Cell\;viability=\frac{Live\;cell\;ssurface}{Scaffold\;surface}\times 100$$

Finally, the scaffold sections were stained by immunohistochemical staining with actin antibody to smooth muscle (Actin, Smooth Muscle (1A4) Mouse Monoclonal Antibody, Cell Marque). This allowed identification of specific protein binding, enabling evaluation of muscle cell differentiation and cytoskeleton organization.

### 2.6. Statistical Analysis

Statistical inference was performed by analyzing the normality of the samples using the *t*-test for independent samples or the Mann–Whitney U test when the distribution was not normal. For comparison of more than two samples, samples with normal distribution were analyzed using the ANOVA test, while the Kruskal–Wallis test was used for samples with non-normal distribution. A statistically significant value was established at *p* < 0.05. For this, the IBM^®^ SPSS^®^ Statistics 19 package was used for statistical analysis.

## 3. Results

### 3.1. Rheological and Morphological Properties of PCL Supports

**Figure 1:**
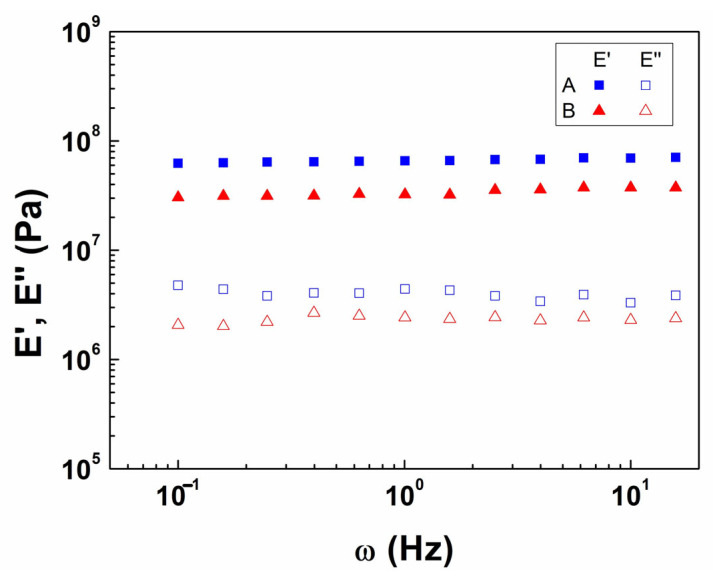
Frequency sweeps (evolution of the elastic modulus (E′) and the viscous modulus (E″)) of the PCL supports with different configurations (designs A and B).

Figure 1 presents the results of the frequency sweep tests for the PCL supports with configurations A (20 × 10 mm with 3 × 10 mm jaws) and B (20 × 5 mm with 3 × 5 mm jaws). In both cases, E′ exhibited an independent behavior of frequency, indicating a predominantly elastic response and excellent mechanical stability, which is typically found in solid-like materials (E′ > E″) [68,69]. Thereby, comparing both configurations, design A displayed higher values of E′ and E″ than design B, showing a stiffer and slightly more energy-dissipative structure. 

Functionally, both configurations satisfy the mechanical requirements for use in dynamically stimulated bioreactors. Specifically, a high value of E′ ensures the effectiveness in the transmission of mechanical stresses to the scaffold, whereas the independence of the applied frequency indicates mechanical stability under cyclic loading conditions (~5 Hz), which is essential for SM stimulation [64,70,71]. Comparing the rheological properties of the two configurations, configuration B exhibits greater deformability (lower stiffness than configuration A). Furthermore, configuration B presents a geometry that enhances adhesion between PCL and the sacrificial matrix (lowering the intermediate void, as shown in Figure S1), promoting more stable deposition of the gelatin matrix and the bioink printing. In fact, this upgraded interfacial integration is critical to maintaining structural fidelity during fabrication. Therefore, based on this combination of adequate mechanical performance and superior processability, configuration B was selected as the reference design for subsequent experiments.

**Figure 2:**
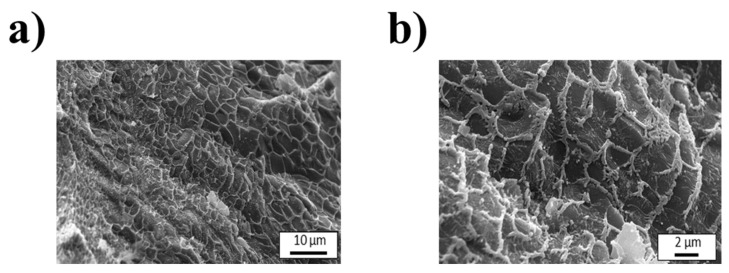
Cryo-SEM micrographs for the support of design B at (**a**) 1500× and (**b**) 5000× magnification.

In the same way, Figure 2 displays cry-SEM micrographs of the PCL support in configuration design B, revealing a homogeneous, well-defined filament arrangement resulting from consistent extrusion and accurate layer-by-layer deposition during the 3D printing process. Particularly, PCL offers the possibility to support the material, as well as hydrophobic characteristics that reduce strong interactions with the sacrificial matrix, facilitating the controlled placement of the bioink during printing and enabling easier separation of the support after the culture process. This behavior is advantageous for systems that require temporary structural guidance without permanent bonding [72,73,74].

### 3.2. Rheological and Morphological Properties of the GE Matrix

The viscoelastic parameters obtained for GE matrices at different concentrations (0.75, 1.5 and 3.0 wt%) are summarized in Table 1, which includes the storage modulus (G′_1_), loss modulus (G″_1_) at 1 Hz, and the critical strain (γ_c_) derived from strain sweep tests.

In this case, G′_1_ and G″_1_ values increased while increasing the gelatin concentration, reflecting a reinforcement of the hydrogel network. Specifically, the 3.0 wt% GE matrix demonstrated the highest value for the viscoelastic parameters, followed by 1.5 and 0.75 wt%, with a consistent trend with previous studies that showed an enhancement in the intermolecular interactions and network density when increasing the biopolymer concentration [75,76,77]. In contrast, the critical strain (γ_c_) did not follow a monotonic trend with concentration. The 1.5 wt% GE matrix exhibited the highest γ_c_ value, slightly exceeding that of the 3.0 wt% system, suggesting that intermediate concentrations may provide an optimal balance between network connectivity and flexibility. At higher concentrations, increased network rigidity may limit deformability, while at lower concentrations, insufficient chain interactions hinder the formation of a stable network [17,78]. Similar non-linear trends have been reported in protein-based hydrogels due to physical crosslinking density and protein–protein interactions [78].

**Figure 3:**
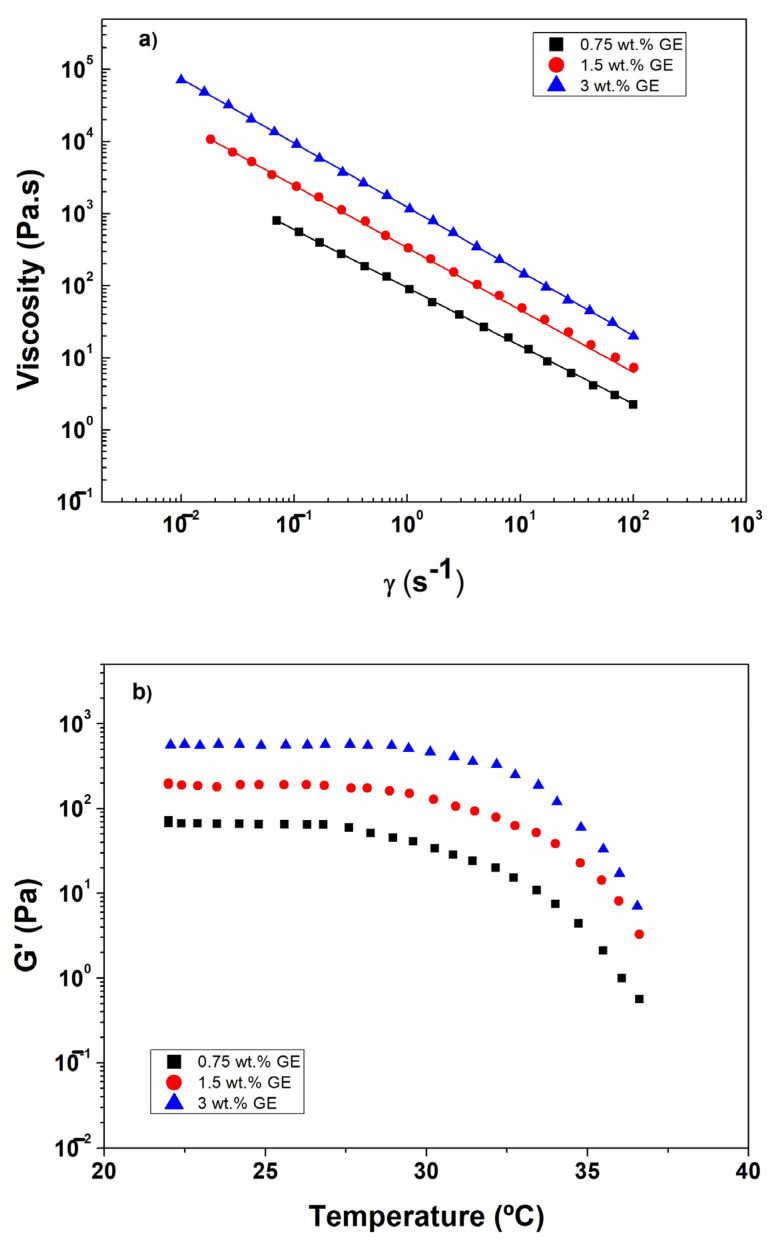
Behavior of GE matrices at: (**a**) flow curves and (**b**) temperature ramps.

The flow behavior of the GE matrices is shown in Figure 3a. All systems exhibited a pronounced shear-thinning (pseudoplastic) behavior, characterized by a decrease in viscosity with increasing shear rate, which is essential for extrusion-based bioprinting, as it enables easy flow through the nozzle under shear while allowing rapid viscosity recovery after deposition, ensuring structural fidelity.

The viscosity data were successfully fitted using the Ostwald–de Waele model [79], and the corresponding parameters are reported in Table 1. The consistency index (K) increased significantly with gelatin concentration, reflecting the higher resistance to flow in concentrated systems. Conversely, the flow behavior index (n) decreased with increasing concentration, confirming a more pronounced non-Newtonian character at higher GE contents. These results are consistent with previous reports on biopolymer-based hydrogels [80,81,82]. From a processing standpoint, viscosity is a critical factor in determining printability. The 1.5 and 3.0 wt% GE matrices exhibited viscosity values within the optimal range for stable filament formation and deposition, whereas the 0.75 wt% system may be too fluid to maintain structural integrity during printing [83,84,85,86,87].

Temperature ramp tests (Figure 3b) revealed a progressive decrease in G′ with increasing temperature, reflecting the thermal weakening of the protein network. Two distinct regimes were observed: an initial gradual decrease in modulus followed by a sharper drop, corresponding to the disruption of the physical crosslinks responsible for gel stability. Matrices with higher gelatin content (1.5 and 3.0 wt%) exhibited a shift of the critical temperature range toward higher values (Table 1), indicating improved thermal stability. However, none of the systems maintained their structural integrity above 35 °C, confirming their suitability as sacrificial materials that can be removed under physiological conditions, which is required for enabling bioink stabilization followed by matrix liquefaction during incubation [88].

Considering the combined effects of viscoelastic properties, deformability, printability, and thermal response, the 1.5 wt% GE matrix was identified as the optimal formulation. This system provides sufficient mechanical support during printing, the highest resistance to structural failure (γ_c_), and adequate thermal sensitivity for subsequent removal, being selected as the reference matrix.

**Figure 4:**
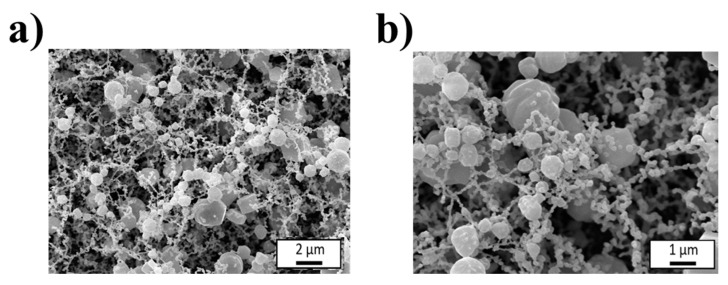
Cryo-SEM images for 1.5 wt% GE reference matrix at (**a**) 5000× and (**b**) 10,000× magnification.

The microstructure of the selected 1.5 wt% GE matrix was further analyzed by cryo-SEM (Figure 4), revealing a heterogeneous network composed of aggregated domains interconnected by fibrillar structures, typically found in physical crosslinked gelatin gels, consistent with the relatively low viscoelastic moduli observed, which explains the temperature-sensitive behavior of the matrix. Upon heating from 22 to 37 °C, the disruption of these weak interactions leads to rapid loss of structural integrity and complete dissolution of the network [66].

### 3.3. Rheological and Morphological Properties of Bioinks

**Figure 5:**
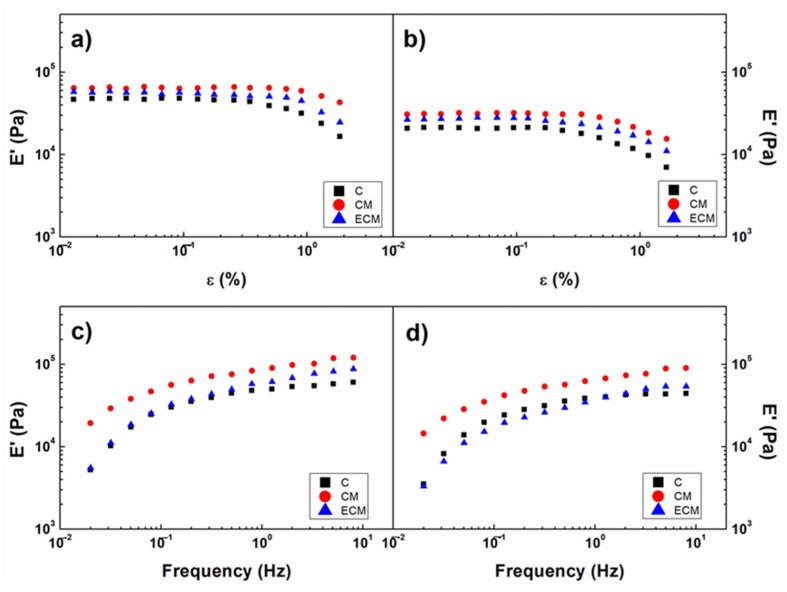
Strain sweep tests performed at (**a**) bioprinting temperature (22 °C) and (**b**) application temperature (37 °C). Frequency sweep tests at (**c**) bioprinting temperature (22 °C) and (**d**) application temperature (37 °C) for the different collagen-based processed bioinks (C, CM and ECM).

Figure 5a,b present the strain sweep results obtained at the bioprinting temperature (22 °C) and physiological temperature (37 °C) for the different collagen-based bioinks (C, CM, and ECM). In all cases, the materials exhibited a typical viscoelastic response, characterized by a linear elastic region followed by a nonlinear regime. The transition between these regions defines the γ_c_, beyond which structural disruption of the hydrogel network occurs [89,90].

A comparison of γ_c_ values (Table 2) reveals that the CM bioink exhibited the highest resistance to deformation at both temperatures, indicating a more robust network capable of sustaining higher mechanical stresses before yielding. The ECM formulation showed intermediate behavior, while the C system displayed the lowest structural stability. These differences can be attributed to the increased complexity of the polymeric network in CM and ECM systems, where additional components reinforced the structure.

The frequency sweep results (Figure 5c,d) provide further insight into the viscoelastic behavior of the bioinks. At 22 °C (Figure 5c), all formulations exhibited a dependence of the E′ on frequency, indicative of weak gel behavior, suggesting a not fully crosslinked network that is dominated by reversible physical interactions (advantageous for extrusion-based bioprinting, as it allows flow under shear while maintaining structural integrity after deposition) [91]. CM and ECM bioinks exhibited higher E′ values, as mentioned above. At 37 °C (Figure 5d), a more pronounced frequency dependence of E′ was observed for all systems, indicating increased structural instability under physiological conditions as a result of a partially weak network.

To enable a quantitative comparison, the viscoelastic parameters at 5 Hz (E′_5_ and η*_5_) were analyzed (Table 2), as this frequency corresponds to the mechanical stimulation conditions applied in the bioreactor. At 22 °C, the CM bioink exhibited the highest E′_5_ and γ_c_ values, balancing stiffness with deformability, while the ECM system showed the highest η*_5_, indicating greater resistance to flow and an increase in viscous resistance. At 37 °C, all bioinks exhibited decreased viscoelastic properties, reflecting thermal softening of the network. However, the relative behavior between formulations remained consistent, with CM maintaining the highest structural stability.

To further quantify the effect of temperature, the parameter β was defined as the ratio of E′_5_ at 37 °C to that at 22 °C for each formulation (Equation (3)). As shown in Table 2, the CM bioink exhibited the highest β value, indicating the lowest sensitivity to temperature changes and therefore greater thermal stability. In contrast, the ECM formulation showed the largest decrease in mechanical properties (β ≈ 0.67), reflecting a more temperature-sensitive network.

(3) $$\beta =\frac{{{iE}^{\prime }}_{5}T\;37\;{}^{\circ}C}{i{E^{\prime }}_{5}T\;22\;{}^{\circ}C}$$

Despite these differences, all bioinks exhibited viscoelastic properties within a suitable range for extrusion-based bioprinting and mechanical stimulation, combining shear-thinning behavior, an adequate elastic modulus and sufficient critical strain [18,70,92], not being a limiting factor in the selection of the bioink.

**Figure 6:**
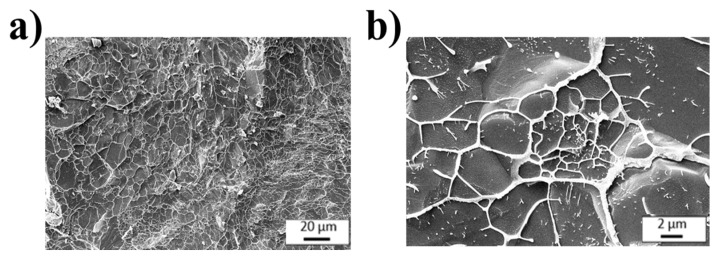
Cryo-SEM images for the ECM bioinks at (**a**) 500× magnification and (**b**) 2000× magnification.

With respect to the morphological properties, the microstructural appearance of ECM-based scaffolding (Figure 6) showed a 3D porous network with a heterogeneous pore size distribution. Moreover, porosity is directly linked to nutrient diffusion, waste removal and cell migration throughout the scaffold. In this context, the ECM bioink exhibits structural features that are highly favorable for SMTE, including high porosity and effective pore interconnectivity.

Hence, considering the combined rheological and morphological results, the ECM formulation is a suitable candidate for TE applications, as it provides a biomimetic microenvironment and adequate mechanical and processing properties.

### 3.4. Biological Evaluation of Bioinks

**Figure 7:**
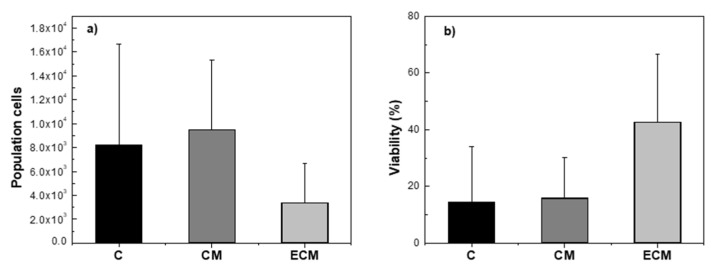
(**a**) Sample cell population and (**b**) cell viability studies for C, CM and ECM scaffolds.

Figure 7 summarizes the cell population (Figure 7a) and viability (Figure 7b) obtained for the different bioink formulations after 4 weeks of culture. Quantitatively, the C and CM bioinks exhibited higher cell population values (8199.29 ± 8464.64 and 9486.43 ± 5846.84, respectively) compared to the ECM formulation (3394.43 ± 3283.48). However, no statistically significant differences were observed between groups (*p* = 0.186), indicating a high variability within the samples. Such variability, although inherent to complex 3D cell-laden systems, suggests that further optimization of bioink formulation and culture conditions is required to improve reproducibility.

**Figure 8:**
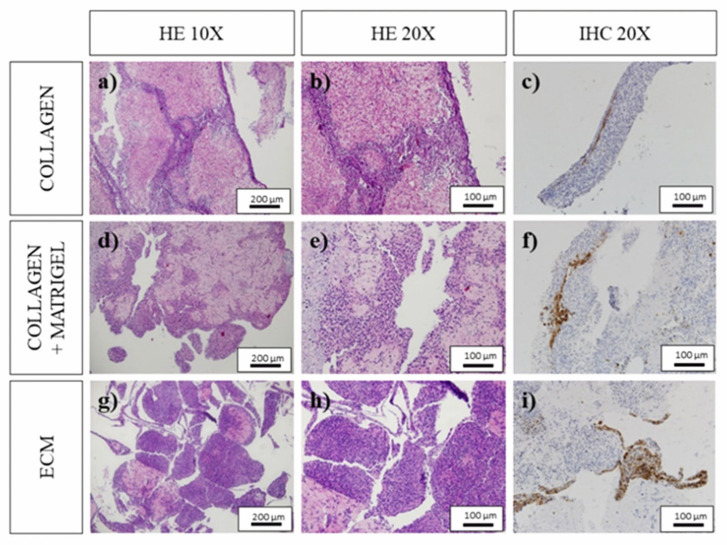
Results of H&E staining and IHC labeling. (**a**) H&E staining of C scaffolds at 10×, (**b**) H&E staining of C scaffolds at 20×, (**c**) results of IHC labeling for studying the actin expression of the C scaffolds at 20×, (**d**) H&E staining of CM scaffolds at 10×, (**e**) H&E staining of CM scaffolds at 20×, (**f**) results of IHC labeling for studying the actin expression of the CM scaffolds at 20×, (**g**) H&E staining of ECM scaffolds at 10×, (**h**) H&E staining of ECM scaffolds at 20× and (**i**) results of IHC labeling for studying the actin expression of the ECM scaffolds at 20×.

Despite the higher cell counts in the C and CM systems, qualitative analysis of the histological images (Figure 8) revealed important differences in cell distribution and viability. In both C (Figure 8a,b) and CM (Figure 8d,e) scaffolds, viable cells were predominantly located at the periphery, while the central regions exhibited large necrotic areas. This spatial distribution suggests limitations in nutrient diffusion and mass transport within these denser hydrogel networks. Consistent with these observations, cell viability in C and CM bioinks was low, at 14.45 ± 19.71% and 15.97 ± 14.24%, respectively. In contrast, the ECM-based scaffolds showed a markedly higher average viability (42.96 ± 23.94%), although variability remained high and differences were not statistically significant (*p* = 0.086).

Morphological analysis of ECM scaffolds (Figure 8g,h) revealed a more heterogeneous cellular distribution, with regions of high cell density coexisting with necrotic zones. Notably, viable cells were frequently associated with filament-like structures within the scaffold, suggesting that local microstructural features may promote improved cell survival. This behavior is consistent with the enhanced porosity and interconnectivity previously observed in ECM-based scaffolds, which likely facilitate better nutrient transport and waste removal. Overall, these results highlight that cell population alone is not a sufficient indicator of scaffold performance, as higher cell numbers in C and CM systems did not translate into improved viability. Instead, the ECM formulation appears to provide a more favorable microenvironment for cell survival, despite lower initial cell density. Regarding cytoskeletal organization, immunohistochemical (IHC) analysis using smooth muscle actin staining (Figure 8c,f,i) revealed limited expression across all formulations. In C scaffolds, weak staining was observed in approximately 5% of viable cells in 50% of the samples, while 25% showed no detectable labeling. Similar results were obtained for CM scaffolds, where staining did not exceed 10% of viable cells and was absent in half of the samples.

ECM scaffolds exhibited slightly improved staining, with approximately 10% of the scaffold area showing positive labeling in 50% of the samples, primarily localized in filamentous regions and scaffold edges. However, a significant fraction of samples still showed minimal or no staining. These results suggest that, although some degree of cytoskeletal organization is present, the overall level of differentiation remains limited under the tested conditions. It is important to note that smooth muscle actin is not a specific marker for skeletal muscle differentiation, and therefore these findings should be interpreted as indicative of early structural organization rather than definitive myogenic maturation.

Taken together, the biological results indicate that while all bioinks are capable of supporting cell survival to some extent, the ECM formulation provides a more favorable balance between structural properties and biological performance. Nevertheless, the high variability and lack of statistical significance highlight the need for further optimization of both bioink composition and culture conditions, particularly in terms of enhancing nutrient transport and promoting cell differentiation.

## 4. Conclusions

This study demonstrates the feasibility of developing a multi-component 3D bioprinted platform integrating mechanical support, a sacrificial matrix and bioactive hydrogels for skeletal muscle tissue engineering applications.

Mechanically stable architectures capable of withstanding cyclic loading and transmitting mechanical stimuli to embedded cells were assessed by the employment of PCL as structural support during the fabrication procedure. This material provided adequate interfacial integration with the sacrificial matrix and bioinks, ensuring structural integrity throughout the fabrication process.

Moreover, gelatin enables controlled and homogeneous bioink deposition, highlighting its crucial role as a sacrificial matrix due to thermoresponsive behavior that eases the transition between a supported printing environment and a free hydrogel construct, being stable at room temperature while quickly dissolving at physiological temperatures. Particularly, the optimal formulation incorporating 1.5 wt% gelatin in the matrix demonstrated an ideal balance of mechanical stability, printability and thermal responsiveness.

Collagen-based bioinks, developed for extrusion bioprinting, exhibited adequate rheological properties (i.e., shear-thinning behavior, suitable viscoelasticity, and resistance to deformation under dynamic conditions), ensuring the processability during the printing process and the structural stability under subsequent mechanical stimulation.

Biologically, the ECM-based bioink demonstrated increased cell viability and spatial cell distribution in comparison to the unitary formulations, without significant differences observed. Thereby, an improvement in the biochemical complexity and an enhancement in the microstructure contribute to the formation of a more auspicious cellular microenvironment.

Overall, the combination of ECM bioink, PCL support, and gelatin-based sacrificial matrix depicted a hopeful method for the production of constructs that are mechanically functional and biologically active. Further optimization of bioink composition, scaffold architecture and culture conditions is needed for the enhancement of the biological outcomes (i.e., the enhancement of cell viability and the promotion of myogenic differentiation). This work provides a versatile and modular platform that integrates mechanical and biological design criteria, contributing to the advancement of 3D bioprinting strategies for skeletal muscle tissue engineering.

## Figures and Tables

**Table 1:** Values of the elastic modulus (G′_1_), viscous modulus (G″_1_), critical strain (ɣ_c_), consistency index (K), rheological behavior index (n) and critical temperature (critical T) for the different GE-based matrices.

Systems	0.75 wt.% GE	1.5 wt.% GE	3 wt.% GE
G′_1_ (Pa)	80.14 ^a^	279.37 ^b^	737.1 ^c^
G″_1_ (Pa)	1.65 ^A^	2.38 ^B^	2.92 ^C^
ɣ_c_ (%)	0.22 ^α^	0.37 ^β^	0.31 ^γ^
K (Pa.s^n^)	95.96 *	355.13 **	1227.61 ***
n	0.19 ^I^	0.15 ^II^	0.11 ^III^
Critical T (°C)	27.5–35	30–35	32.5–35

Note: Different letters/symbols included as superscripts in each row correspond to significantly different values.

**Table 2:** Values of the critical strain (ɣ_c_), elastic modulus (E′_5_), and complex viscosity (η*_5_) at 5 Hz and degree of degradation (β) for the systems prepared with the different compositions: C, CM and ECM for the tests at bioprinting temperature (22 °C) and at the application temperature (37 °C).

**Temperature**	**Compositions**	**ɣ_c_ (%)**	E′ **_5_** **(MPa)**	η*_5_ (Pa·s)	**β**
22 °C	C	0.184 ^a^	0.055 ^A^	1703 ^α^	0.747
	CM	0.336 ^b^	0.118 ^B^	2428 ^β^	0.795
	ECM	0.243 ^c^	0.082 ^C^	3031 ^γ^	0.679
37 °C	C	0.125 ^d^	0.042 ^A^	-	
	CM	0.221 ^c^	0.088 ^C^	-	
	ECM	0.167 ^a^	0.055 ^A^	-	

Note: Different letters/symbols included as superscripts in each column correspond to significantly different values.

## Data Availability

The original contributions presented in this study are included in the article. Further inquiries can be directed to the corresponding author.

## Supplementary Materials

The following supporting information can be downloaded at: https://www.mdpi.com/article/10.3390/polym18101223/s1.
