# Supplementary material for: Multi-Component 3D Bioprinted Platform with Sacrificial Matrix and Collagen-Based Bioinks for Skeletal Muscle Tissue Engineering
Source: Polymers (Basel). 2026 May 17;18(10):1223. doi: 10.3390/polym18101223 (PMC13210422; doi:10.3390/polym18101223)
Supplement: Supplementary file 1 [file polymers-18-01223-s001.zip › polymers-4291754-supplementary.pdf]

Multi-Component 3D Bioprinted Platform with Sacrificial Matrix and Collagen-Based Bioinks for Skeletal Muscle Tissue Engineering

Carmen Mª. Granados-Carrera[1], Francisco José Calero Castro[2, 3], Victor M. Perez-Puyana[4,*], Mercedes Jiménez-Rosado[5], Jaime Navarrete-Damián[6], Fernando de la Portilla de Juan[2, 3], Alberto Romero[1]

SUPPLEMENTARY MATERIAL

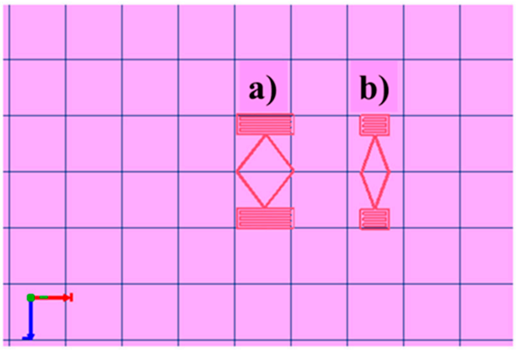

Figure S1. Designs of the 3D PCL holders with different dimensions using FreeCAD 0.17 software: a) 20x10 mm with 3x10 mm clamping jaws and b) 20x5 mm with 3x5 mm clamping jaws.

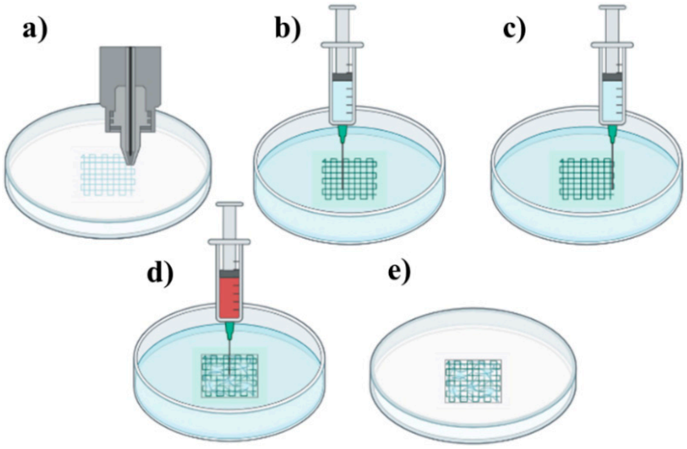

Figure S2. Bioprinting process. a) PCL printing, b) gelatin deposition, c) neutralization of gelatin, d) bioink deposition and e) gelatin liquefaction and bioink crosslinking.
